# Diseases of the Joints and Spine

**Published:** 1896-03

**Authors:** 


**Diseases of the Joints and Spine.** By Howard Marsh. New and Revised Edition. Pp. xvi., 532. London: Cassell and Company, Limited. 1895.

The new edition of this work has been published nine years after the first edition, and so many changes have taken place in our knowledge of joint diseases during this period that the author has done wisely to re-write many chapters, and to make considerable alteration in others, so as to give us now what is almost a new book on the subject. We took occasion to point out in our review of the first edition that the author's strong point was evidently not pathology, and although we notice a great improvement in the present edition, yet the work is still mainly clinical. We are happy, however, to be able to say that from a clinical standpoint the book is excellent. It gives in a very readable manner a somewhat brief, though sufficient, account of the various forms of joint disease, illustrated by numerous cases which have come under the author's observation, while it gives also a slight outline of the pathological changes met with in these various forms of disease.

Mr. Howard Marsh's methods of treatment are eminently conservative, though marked by sound judgment. We are glad to see that credit is given to a Bristol surgeon (Mr. F. R. Cross) for the first suggestion of erasion of joints for tuberculous disease. We notice, however, that, like most other surgeons of experience, the author takes a very doubtful view of the value of this operation, except in a few selected cases, and he is rather disposed to limit its usefulness to the elbow and ankle. Two chapters are introduced giving a short account of the principal diseases of the spine, treating this subject almost from a clinical point of view: this will distinctly add to the value of the book; for the author's experience in the management and treatment of the various forms of spinal disease has been very extensive, and he speaks with authority upon this subject. The book is eminently readable, being well written and illustrated.

